# Novel CSF1R-positive tenosynovial giant cell tumor cell lines and their pexidartinib (PLX3397) and sotuletinib (BLZ945)-induced apoptosis

**DOI:** 10.1007/s13577-022-00823-0

**Published:** 2022-12-02

**Authors:** Suyanee Thongchot, Supani Duangkaew, Wasan Yotchai, Sorranart Maungsomboon, Rapin Phimolsarnti, Apichat Asavamongkolkul, Peti Thuwajit, Chanitra Thuwajit, Chandhanarat Chandhanayingyong

**Affiliations:** 1 Department of Immunology, Faculty of Medicine Siriraj Hospital, Mahidol University, Bangkok, Thailand; 2 Siriraj Center of Research Excellence for Cancer Immunotherapy, Research Department, Faculty of Medicine Siriraj Hospital, Mahidol University, Bangkok, Thailand; 3 Division of Musculoskeletal Oncology, Department of Orthopaedic Surgery, Faculty of Medicine, Siriraj Hospital, Mahidol University, 2 Wang Lang Road, Bangkoknoi, Bangkok, 10700 Thailand; 4 Department of Pathology, Faculty of Medicine Siriraj Hospital, Mahidol University, Bangkok, Thailand

**Keywords:** Tenosynovial giant cell tumor, Pigmented villonodular synovitis, CSF1R, Pexidartinib, Sotuletinib

## Abstract

Tenosynovial giant cell tumor (TGCT) is a mesenchymal tumor derived from the synovium of the tendon sheath and joints, most frequently in the large joints. The standard of care for TGCTs is surgical resection. A new targeting approach for treating TGCTs has emerged from studies on the role of the CSF1/CSF1 receptor (CSF1R) in controlling cell survival and proliferation during the pathogenesis of TGCTs. We established four novel cell lines isolated from the primary tumor tissues of patients with TGCTs. The cell lines were designated Si-TGCT-1, Si-TGCT-2, Si-TGCT-3, and Si-TGCT-4, and the TGCT cells were characterized by CSF1R and CD68. These TGCT cells were then checked for cell proliferation using an MTT assay and three-dimensional spheroid. The responses to pexidartinib (PLX3397) and sotuletinib (BLZ945) were evaluated by two-dimensional MTT assays. All cells were positive for α‑smooth muscle actin (α‑SMA), fibroblast activation protein (FAP), CSF1R, and CD68. Except for Si-TGCT-4, all TGCT cells had high CSF1R expressions. The cells exhibited continuous growth as three-dimensional spheroids formed. Treatment with pexidartinib and sotuletinib inhibited TGCT cell growth and induced cell apoptosis correlated with the CSF1R level. Only Si-TGCT-4 cells demonstrated resistance to the drugs. In addition, the BAX/BCL-2 ratio increased in cells treated with pexidartinib and sotuletinib. With the four novel TGCT cell lines, we have an excellent model for further in vitro and in vivo studies.

**Supplementary Information:**

The online version contains supplementary material available at 10.1007/s13577-022-00823-0.

## Introduction

Tenosynovial giant cell tumors (TGCTs), formerly termed pigmented villonodular synovitis, are neoplasms that develop in the synovium of joints, tendon sheaths, and bursae. TGCTs are present in two types. The predominant form is diffuse and encompasses the entire synovium, whereas the minor type is localized, involving only a portion of the synovium [1]. Typically, TGCTs are found in 20–50-year-old patients, with an approximately equal distribution between men and women [2]. However, the diffused form is more common among young women [3]. TGCT tissue comprises many cell types. They include fibroblast-like synovial cells, sideroblasts, foam cells, histiocyte-like cells, hemosiderin-laden macrophages, and multinucleated giant cells [1, 4]. In addition, a small proportion of TGCT cells make up a neoplastic clone that expresses colony-stimulating factor 1 (CSF1). Approximately one-third of TGCT cases have a t (1;2) translocation linking the COL6A3 gene on chromosome 2q35 and the *CSF1* gene on chromosome 1p13 [5, 6]. High levels of CSF1 expression in TGCTs result from this *COL6A3-CSF1* fusion [6, 7]. Consequently, the underlying cause of TGCTs can be targeted by inhibiting signaling between CSF1 and the CSF1 receptor (CSF1R) [8, 9]. Research has shown that the monocyte–macrophage lineage marker CD68 stains synovial-lining cells; double staining revealed that CD68 is also expressed by TGCT cells that express CSF1 [10]. Other work found high CD68 expression levels in several tumor types, particularly TGCTs, compared with normal tissue samples [11].

The current standard treatment for TGCTs includes arthroscopic or open synovectomy. However, the diffused TGCT is more difficult to resect. It also has a 20% to 55% chance of local recurrence, resulting in joint destruction requiring joint replacement or amputation [3, 12]. Besides surgery, the CSF1R inhibitor pexidartinib and the monoclonal antibody emactuzumab are used to treat TGCTs [13, 14].

Pexidartinib (PLX3397) is a small-molecule tyrosine kinase inhibitor that targets *CSF1R*, *KIT* (KIT proto-oncogene, receptor tyrosine kinase), and *FLT3* (FMS-like tyrosine kinase 3) harboring an internal tandem duplication (ITD) mutation [15, 16]. Overexpression of the CSF1R ligand promotes cell proliferation and accumulation in the synovium [17]. In vitro studies revealed that the growth of osteosarcoma cell lines that depended on CSF1R and the receptor’s ligand-induced autophosphorylation property was inhibited by pexidartinib [18]. Pexidartinib is the first drug approved by the United States Food and Drug Administration for treating adult patients with TGCTs who have severe morbidity or functional limitations that are not amenable to surgery [15]. A study showed that in 62% of patients taking pexidartinib for 38 month median follow-up, TGCTs shrank by 30% or more, resulting in pain relief and less stiffness [16, 19]. However, a subset of patients did not respond to pexidartinib. Side effects including lightening of hair color, fatigue, and reversible hepatotoxicity meant the drug is not appropriate for all patients with TGCTs [20]. There is a need for effective and less harmful treatment for TGCTs. Sotuletinib (BLZ945) is a small-molecule inhibitor that inhibits CSF1R and is being tested in a clinical phase II trial (NCT04066244).

Having TGCT cell lines would facilitate studies of the pathological interactions between the cell components of TGCTs, leading to alternative treatment approaches. In the present study, we established and characterized novel TGCT cell lines, designated Si-TGCT-1–4, from surgically resected tumor tissues. To demonstrate the usefulness of these cells, we studied their proliferation and spheroid formation characteristics and examined their responses to two CSF1R inhibitors: pexidartinib and sotuletinib. The results revealed that all Si-TGCT-1–4 cells can be used in preclinical research and the last one, Si-TGCT-4, can be used especially on TGCT drug resistance.

## Materials and methods

### Patient background

The research protocol was evaluated and approved by the Siriraj Institutional Review Board (Si894/2020). Written informed consent was obtained from participants before their enrollment between November 2020 and November 2021. The patients’ demographic data is detailed in Table 1.

**Table 1:** Clinical characteristics and demographic data of the patients with TGCT

Patient ID	Characteristics						
	Age (years)	Sex	Location	Type	Treatment	Recurrent	Outcome
Si-TGCT-1	40	Male	Knee	Localized	Resection	–	NED
Si-TGCT-2	70	Male	Ankle	Localized	Resection	–	NED
Si-TGCT-3	44	Female	Hip	Diffused	Resection	1	AWD
Si-TGCT-4	38	Male	Knee	Diffused	Resection	2	AWD

*AWD* active with disease, *NED* no evidence of disease

### Cancer cell isolation and culture

TGCT cancer tissues were obtained from 4 patients who underwent surgery at Siriraj Hospital, Bangkok, Thailand. The samples were designated Si-TGCT-1, Si-TGCT-2, Si-TGCT-3, and Si-TGCT-4. A fresh TGCT tissue sample (1 × 1 × 1 cm^3^) was isolated from TGCT tissue and surgically resected according to our previous guidelines [21]. Briefly, TGCT tissues were incubated in a 10X antibiotic mixture (1 U/ml penicillin G sodium and 1 mg/ml streptomycin; Thermo Fisher Scientific Inc.), diluted in DMEM/F12. The obtained tissue was minced into 0.1 × 0.1 × 0.1 cm^3^ sections and incubated with an enzyme cocktail mix (Miltenyi Biotec GmbH) for 1 h at 37 °C. The digested cells were passed through a 70 μm nylon filter (SPL Life Sciences) and cultured in DMEM/F-12 media (Gibco BRL) supplemented with 10 ng/ml of epidermal growth factor (EGF, PeproTech Inc.), 5 μg/ml insulin (Sigma‑Aldrich; Merck KGaA), 0.32 μg/ml hydrocortisone (Sigma‑Aldrich; Merck KGaA) and 10 μM ROCK inhibitor (Y27632, StemMACS, Miltenyi Biotec GmbH). The attached cells were sub-passaged continuously, periodically checked for negative mycoplasma, and stored in liquid nitrogen.

The commercial human breast cancer cell line MDA‑MB‑231 and the human choriocarcinoma cell line Bewo (purchased from American Type Culture Collection, ATCC) were cultured at 37 °C in a humidified atmosphere of 5% CO_2_ in Dulbecco’s modified Eagle’s (DMEM) and DMEM/F-12 media (Gibco, Thermo Fisher Scientific Inc.) supplemented with 10% fetal bovine serum (FBS; v/v; Gibco, Thermo Fisher Scientific Inc.), 100 U/ml penicillin, and 100 μg/ml streptomycin (both from Sigma‑Aldrich; Merck KGaA). MDA‑MB‑231 was used as the CSF1R-negative control, and Bewo was used as the CSF1R-positive control.

### Detection of markers by immunofluorescence staining

For IF staining, cells at 1 × 10^4^ were seeded on sterile glass coverslips for 24 h, fixed in cold absolute methanol, and subjected to staining with antibodies against epithelial cytokeratin (CK)‑19 and pan CK. Specific markers for stromal fibroblasts—α‑SMA and FAP—were used for quality control of the cancer cell purity. CSF1R was evaluated in the obtained TGCT cells. Briefly, cells were permeabilized with 0.2% Triton‑1X PBS and incubated overnight at 4 °C with the following primary antibodies: mouse anti‑human panCK antibody (sc‑8018; Santa Cruz Biotechnology Inc.); mouse anti‑human CK‑19 antibody (Santa Cruz Biotechnology Inc.); mouse anti‑human α‑SMA antibody (Sigma‑Aldrich; Merck KGaA); rabbit anti‑human fibroblast activation protein (FAP) antibody (ab53066; Abcam); and rabbit anti‑human CSF-1R antibody (ab205921; Abcam). The goat anti‑mouse IgG‑Cy3 antibody (#115‑166‑071; Jackson ImmunoResearch Laboratories Inc.) or the donkey anti‑rabbit IgG (H + L) highly cross‑adsorbed secondary antibody Alexa Fluor 488 (21,206; Thermo Fisher Scientific Inc.) was used. The nuclei were stained with Hoechst 33,342 (Invitrogen; Thermo Fisher Scientific Inc.). Fluorescence was captured with a ZEISS LSM 800 confocal laser fluorescence scanning microscope (Axio Observer 7 LSM 800; Zeiss GmbH).

### Western blot analysis

Cell pellets were resuspended in a cell lysis buffer (Cell Signaling Technology Inc.). After centrifugation, the protein content of the supernatants was determined using a Bradford Protein Assay Kit (Bio‑Rad Laboratories Srl.). Then, 60 mg of protein was prepared in a sample buffer containing 10% SDS, 1.0 M Tris-HCl pH 6.8, 8% glycerol, and 0.05% (w/v) bromophenol blue. Proteins were separated by sodium dodecyl sulfate–polyacrylamide gel electrophoresis (SDS–PAGE) and transferred to a polyvinylidene difluoride (PVDF) membrane. The membranes were incubated overnight with the following primary antibodies: CSF1R (ab205921; Abcam); BAX (mouse anti‑human BAX antibody; 610,983; Becton Dickinson Holdings Pte. Ltd.); BCL‑2 (rabbit anti‑human BCL‑2 antibody; ab196495; Abcam); and β‑actin (mouse anti-β‑actin antibody; sc‑47,778; Santa Cruz Biotechnology Inc.). Further incubation was undertaken with horseradish peroxidase (HRP)-conjugated secondary antibodies for 1 h. The signals were visualized by ECL (Thermo Fisher Scientific Inc.) under Gel Document Syngene (Syngene), and the bands were quantified by ImageJ (version 1.48v; National Institutes of Health, Bethesda, MD, USA). The densitometric values of all protein bands were normalized to that of β-actin and quantified using ImageJ (version 1.52a).

### CD68 detection by flow cytometry

TGCT cells (5 × 10^5^) were harvested and blocked in 5% FBS in PBS 1X before incubation for 30 min with PE-conjugated monoclonal antibody Y1/82A human CD68 (21,270,684; ImmunoTools GmbH) or the isotype control (PE-conjugated mouse IgG1 isotype control; 21,815,014; ImmunoTools GmbH) for 30 min at 4 °C. After 3 washes in 1X PBS buffer, the cells were detected by a CytoFLEX flow cytometer (Beckman Coulter Inc.) and analyzed using CytExpert software (version 2.1; Beckman Coulter Inc.). Monocytes isolated from peripheral blood mononuclear cells and MDA-MB-231 cells were used as positive control cells.

### Genetic analysis

The *COL6A3-CSF1* fusion was examined as previously described [7]. Total RNA was extracted from tumor tissue and cells using the miRNAeasy Mini Kit (Qiagen). Extracted RNA (1 µg) was reverse-transcribed with Superscript III Reverse Transcriptase (Invitrogen; Carlsbad, CA, USA) according to the manufacturer’s instructions. The *COL6A3-CSF1* fusion transcript was amplified with nested PCR using Platinum Taq DNA Polymerase High Fidelity (Thermo Fisher Scientific) with the following primers: first primers, COL6A3_F1 (forward), 5′-CTATTTGCAAGCTGCCAACGCCT-3′; CSF1_R1 (reverse), 5′-TTCCCTCTACACACTGGCAGTTCCACC-3′; and second primers, COL6A3_F2 (forward), 5′-CTAGCCAGGCGAATAAGGGCAGAGC-3′; CSF1_R2 (reverse), 5′-TCTGGTTGCTCCAAGGGAGAATCC-3′.

### Cell proliferation

MTS (3‑[4,5‑dimethylthiazol‑2‑yl]‑5‑[3‑carboxymethoxyphenyl]‑2‑[4‑sulfophenyl]‑2H‑tetrazolium; G3581; Promega) was used to assay cell viability, following the manufacturer’s instructions. Si-TGCT-1–4 cells were plated in triplicate in 96-well plates at 5000 cells/200 μl. Twenty μl of MTS reagent was added to each well at 24, 48, 72, and 96 h. The wells were incubated in a humidified, 5%-CO_2_ atmosphere for a minimum of 2 h. Absorbance at 490 nm was recorded.

### Three-dimensional spheroid formation

In vitro spheroids were obtained. TGCT single-cell suspensions were generated from trypsinized monolayers, with 1000 cells supplemented with 2.5% cold Matrigel (BD Biosciences) in 200 μl of complete DMEM F/12 medium and seeded into pre‑cooled, 96‑well, ultra‑low attachment plates (CLS7007; Costar/Corning Inc.). Centrifugation at 4 °C at 300 × g for 3 min was performed, and the cells were maintained at 37 °C in a humidified 5% CO_2_ atmosphere. The spheroid proliferation rate and size were monitored for up to 10 days under an inverted-light microscope Olympus IX71 using Olympus CellSens standard software.

### Drug cytotoxicity assay

The effects of pexidartinib and sotuletinib on TGCT cell proliferation were assessed. TGCT cells were treated with pexidartinib (0, 0.2, 2, 20, and 200 μM) or sotuletinib (0, 0.1, 1, 10, 100, and 1000 μM) diluted in 10% FBS in DMEM/F-12 medium. The MTS assay and absorbance at 490 nm were performed after an incubation duration of 96 h.

### Statistical analysis

The values are represented as the mean ± standard deviation (SD) from 3 independent assays. All statistical calculations were performed using GraphPad Prism, version 7.04 (GraphPad Software Inc., La Jolla, CA, USA). The data from 2 groups were analyzed by paired Student’s *t*‑tests, and multiple groups were assessed by 1‑way repeated‑measure analysis of variance (ANOVA). The level of statistical significance was set at *P* < 0.05.

### Cell authentication by STR profiling

DNA fingerprint was performed by fluorescent-based PCR technique using capillary electrophoresis at Human Genetic Laboratory, Department of Pathology, Faculty of Medicine Ramathibodi Hospital, Mahidol University, Bangkok, Thailand. Twenty short tandem repeat (STR) loci plus the gender determining locus, Amelogenin, were amplified by six multiplex PCR and separated on ABI 3730XL Genetic Analyzer. The signals were then analyzed by the software GeneMapper [22].

## Results

### Patient backgrounds

**Fig. 1:**
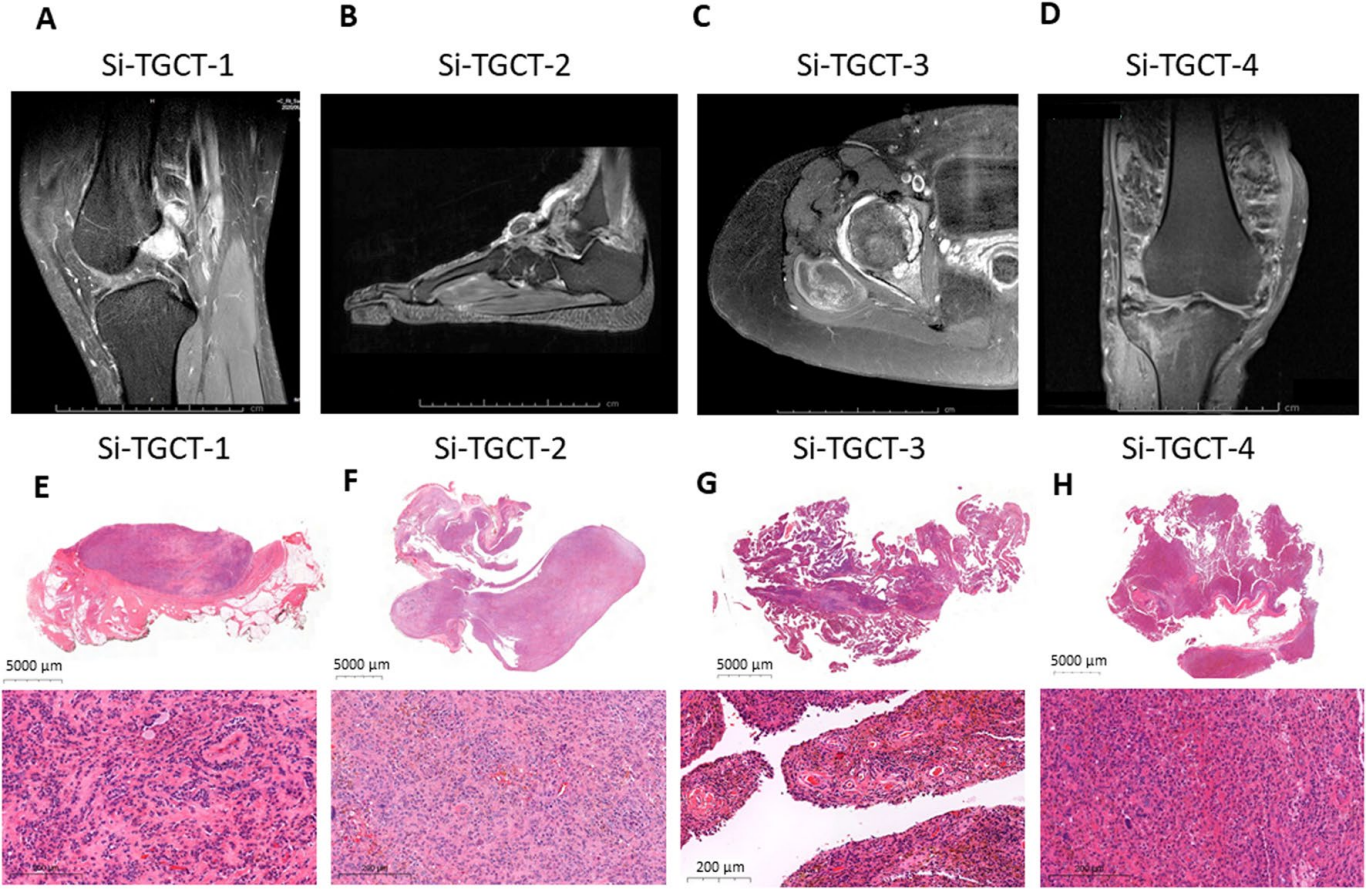
Clinical imaging and pathological diagnoses of 4 patients with TGCT. **A**–**D** Magnetic resonance imaging revealed 2 localized-type TGCTs of the knee and ankle (**A**, **B**) and 2 diffused-type TGCTs of the hip and knee (**C**, **D**). Lesions exhibited areas of low T1-weighted signals with blooming artifacts on gradient echo. **E**–**H** Hematoxylin and eosin staining of TGCTs showing mononuclear stromal cells with stromal fibrosis, including the formation of a hyalinized collagen matrix and a small area of multinucleated osteoclast-like giant cells

The Si-TGCT-1, -2, -3, and -4 cells were retrieved from 4 patients aged between 38 and 70 who had been treated surgically at Siriraj Hospital, Mahidol University (Table 1). Two cases involved diffuse-type TGCTs. The localized type of TGCT was Si-TGCT-1 in the posterior knee and Si-TGCT-2 in the ankle. All patients were treated by synovectomy alone. No adjuvant such as CSF1R inhibitor or radiation was administered. Magnetic resonance imaging revealed joint effusion, hemosiderin deposition, expansion of the synovium, and marginal bony erosion in all four patients (Fig. 1A–D). TGCT tissue showed low signal intensity on T1W and T2W with blooming artifacts on gradient-echo due to iron in hemosiderin. Gross pathology was observed in the operative field and showed proliferative villi extending from the synovium. Hematoxylin and eosin staining (Fig. 1E–H) and a low-power field revealed mononuclear stromal cells infiltrating the synovium. High vascularization of the villi line within plump synovium was evident in hemosiderin-stained multinucleated giant cells, with pigmented foam cells or lipid-laden histiocytes and high mitotic figures.

### Characterization of TGCT cell lines

**Fig. 2:**
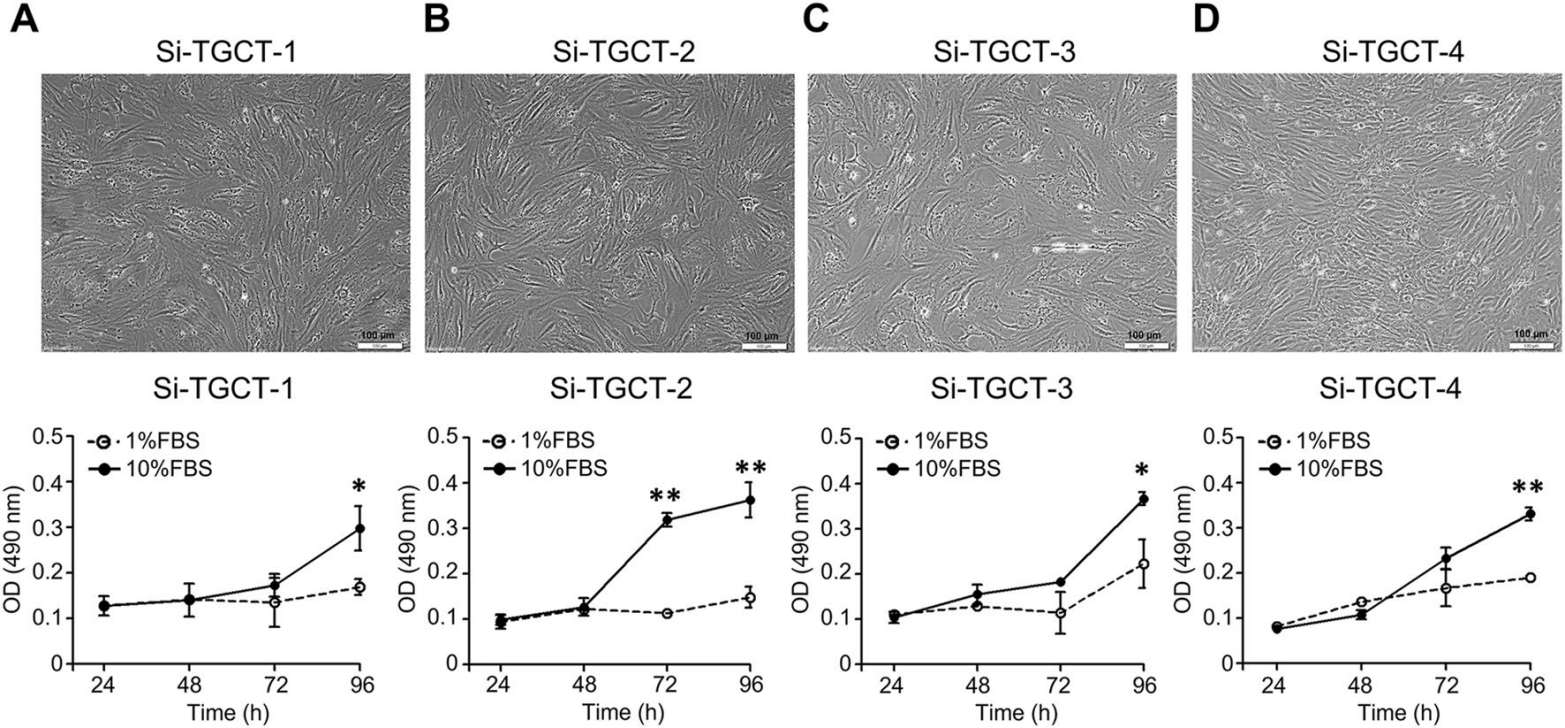
Morphology of the established TGCT cell lines: **A** Si-TGCT-1 (passage 32), **B** Si-TGCT-2 (passage 29), **C** Si-TGCT-3 (passage 23), and **D** Si-TGCT-4 (passage 18). Typical morphology of stable culture cells under a phase-contrast light microscopy (original magnification =  × 100; scale bars = 100 μm). The growth curves were analyzed using the MTS assay at 24, 48, 72, and 96 h normalized with time 0 h, quantified by measuring the absorbance at 490 nm. Statistical significances were set at *P < 0.05; **P < 0.01, compared with a 1% fetal bovine serum culture condition

**Fig. 3:**
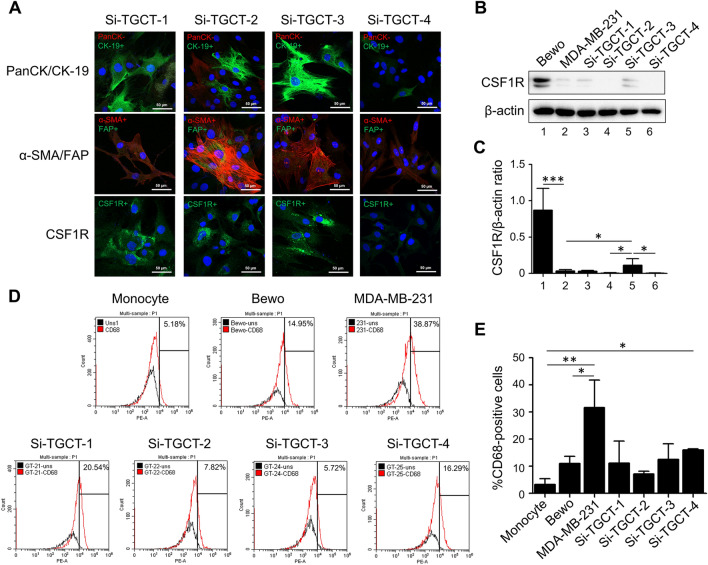
Biological marker detection in the in‑house TGCT cells (Si-TGCT-1, Si-TGCT-2, Si-TGCT-3, and Si-TGCT-4). **A** Immunofluorescence staining, consisting of PanCK (red fluorescence), CK-19 (green fluorescence), α-SMA (red fluorescence), FAP (green fluorescence), and CSF-1R (green fluorescence). Staining with Hoechst33342 (blue fluorescence) was conducted to visualize chromatin; images were captured at × 630 original magnification; scale bars = 50 μm. (Si-TGCT-1: passage 29, Si-TGCT-2: passage 25, Si-TGCT-3: passage 22 and Si-TGCT-4: passage 15). **B** CSF-1R expression by Western blot assay. β‑actin was used as the loading control. **C** Densitometry data of CSF-1R/β‑actin ratio from 3 separate experiments (expressed as mean ± standard deviation) is shown in the histograms. Statistical significances were set at *P < 0.05; ***P < 0.001, compared with negative-control cells, MDA-MB-231. (Si-TGCT-1: passage 12, Si-TGCT-2: passage 4, Si-TGCT-3: passage 16, and Si-TGCT-4: passage 11). **D**, **E** Expression of CD68 positive cells detected by flow cytometry. The percentages of positive cells for the anti-CD68 were compared with an isotype control and are represented in the graph as mean ± SD (isotype control, in black; specific antibodies, in red). *P < 0.05; **P < 0.01, compared with negative-control cells, monocyte isolated from whole blood. (Si-TGCT-1: passage 35, Si-TGCT-2: passage 32, Si-TGCT-3: passage 26, and Si-TGCT-4: passage 21)

The Si-TGCT-1, -2, -3, and -4 cell lines were established from the primary tumor tissues of patients with TGCTs. Three months after the adherent cells were initiated, the cells were maintained in culture through over 30 passages over the following 2 years. Mycoplasma contamination was negative, as no mycoplasma-specific DNA was detected in the cell-conditioned medium via quantitative real-time polymerase chain reaction (data not shown). Under a phase-contrast microscope, all TGCT cells had monotonous, spindle-shaped morphologies (Fig. 2A–D) with different doubling times. No osteoclast-like giant cell was observed. The growth rates of Si-TGCT-1 (localized-type TGCT in the knee) were the slowest of all the diffused TGCT cell line cultures in 10% FBS. The doubling time of Si-TGCT-1 was 78 h, whereas the times for the 3 other cell lines ranged between 45 and 53 h (Table 2; Fig. 2). CK-19, α-SMA, FAP, and CSF1R were present in all TGCT cells. In contrast, PanCK was only present in Si-TGCT-2 cells (Table 2, Fig. 3A). Western blot analysis of CSF1R protein expression was basally expressed in 2 cell lines (Si-TGCT-1, and -3) compared with high expression in Bewo cells (CSF1R-positive control cells) and low CSF1R-expressing MDA-MB-231 negative control cells (Fig. 3B–C). Neither CSF1 expression nor *COL6A3-CSF1* translocation was evident in any of the cell lines (data not shown). The expression of CD68 was confirmed by flow cytometry, and the proportion of CD68-positive cells was significantly higher in the MDA-MB-231-positive control cells (Table 2; Fig. 3D–E). Interestingly, CD68 was positive in all TGCT cells. However, Si-TGCT-4 cells had a significantly higher percentage of CD68 than monocyte negative control cells.

**Table 2:** Characteristics of the established TGCT cell lines

Characteristics	Patient ID			
	Si-TGCT-1	Si-TGCT-2	Si-TGCT-3	Si-TGCT-4
Origin	Primary tumor	Primary tumor	Primary tumor	Primary tumor
Growth characteristics	Adherent	Adherent	Adherent	Adherent
Doubling time (h) in 1% FBS medium	141.92 ± 0.85	271.38 ± 1.53	136.87 ± 1.86	92.60 ± 1.80
Doubling time (h) in 10% FBS medium	78.87 ± 2.39	51.03 ± 3.52	52.89 ± 1.13	45.34 ± 1.17
Sizes of the spheres (× 10^7^ µm^3^)	0.75 ± 0.13	1.88 ± 0.05	0.61 ± 0.16	0.71 ± 0.13
IF for PanCK	–	+	–	–
IF for CK-19	+	+	+	+
IF for α-SMA	+	+	+	+
IF for FAP	+	+	+	+
IF for CSF1	–	–	–	–
IF for CSF1R	+ /High	+ /High	+ /High	+ /Low
FC for CD68	15.54 ± 5.11%	7.82 ± 1.90%	11.72 ± 7.45%	18.29 ± 0.23%

*α-SMA* alpha-smooth muscle actin, *CSF1R* colony-stimulating factor 1 receptor, *FAP* fibroblast activated protein, *FBS* fetal bovine serum, *FC* flow cytometry, *IF* immunofluorescence, *PanCK*, pan-cytokeratin

**Fig. 4:**
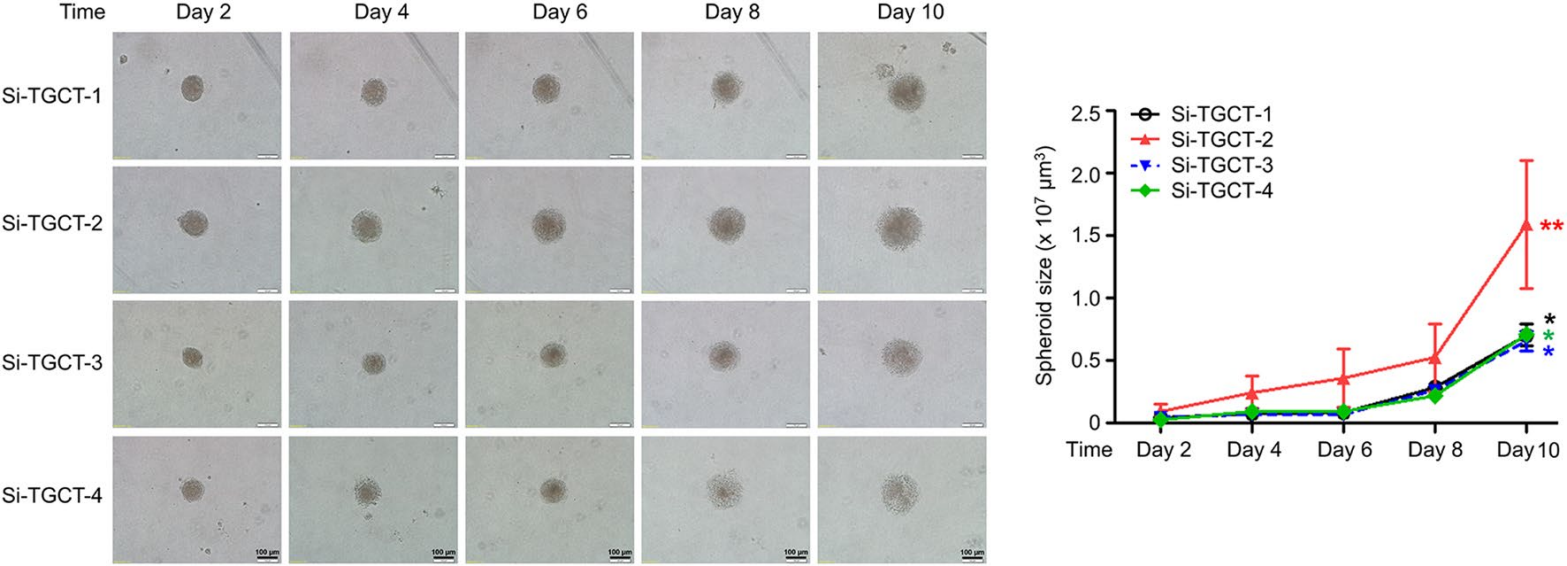
Proliferation ability is shown by 3‑D spheroids on days 2, 4, 6, 8, and 10. The images were captured under light microscopy at × 100 magnification; scale bars, 100 μm. Statistical significances were set at *P < 0.05; **P < 0.01, compared with day 2, normalized with time day 0 of culture time. (Si-TGCT-1: passage 29, Si-TGCT-2: passage 25, Si-TGCT-3: passage 22, and Si-TGCT-4: passage 15)

Si-TGCT-1–4 cells’ ability to form spheroids was observed on low-attachment substrates (Fig. 4). The spheroid outline was less regular and appeared round. To calculate the sphere size, the diameters of at least 10 spheres were measured every second day; the average dimensions of the TGCT spheres are detailed in Table 2. On day 10, all TGCT cells formed larger spheres whose diameters varied from 0.61 × 10^7^ to 1.88 × 10^7^ μm^3^. However, colonospheres from all lines displayed similar morphology. No invasive behavior was observed. Si-TGCT-1–4 cells were immortalized and showed constant growth at their latest passage of 36, 33, 27, and 22, respectively. They were capable of invasion and spheroid formation from the early to the later passage. Tumorigenesis in nude or SCID mice had not been done in this study. Additional *in vivo* experiments using TGCT cell lines are needed for further study.

### Pexidartinib (PLX 3397) and sotuletinib (BLZ 945) sensitization of TGCT cell lines

**Fig. 5:**
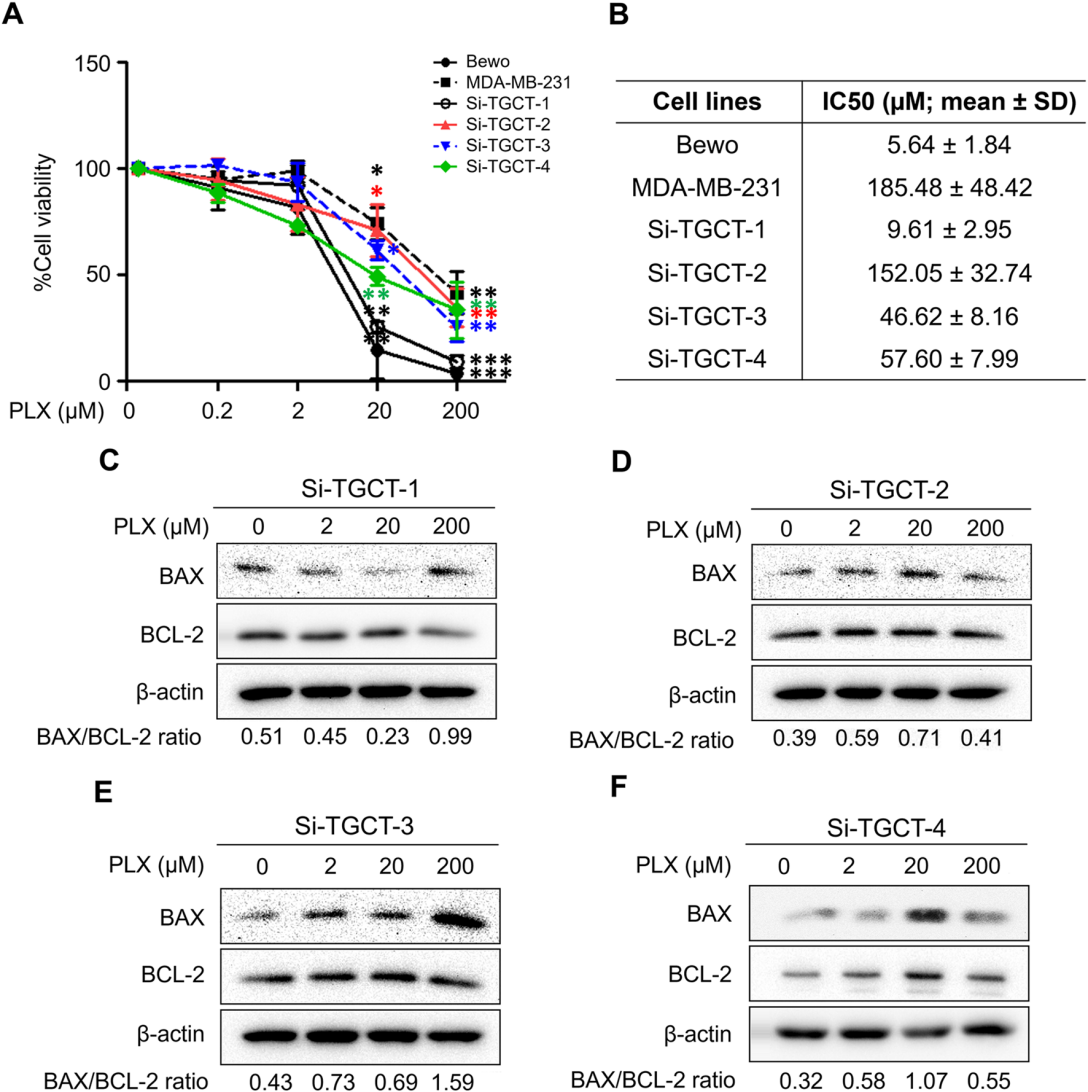
The treatment of pexidartinib (PLX3397) induced cell death in TGCT cells. **A** Cytotoxicity analysis of pexidartinib treatment. Pexidartinib induced cell death in TGCT cells was performed by MTS assay at 0, 0.2, 2, 20, and 200 µM for 96 h. Quantitative results of MTS staining were performed in triplicate; data represented by mean ± SD. **B**–**F** TGCT cell pellets were subjected to check the expression of BAX and BCL‑2 in pexidartinib treatment at 0, 2, 20, and 200 µM for 96 h by Western blot analysis. β‑actin was used as the protein loading control. The ratio of BAX/BCL‑2 was analyzed and reported from the relative band intensity of the Western blotting. *P < 0.05; **P < 0.01 and ***P < 0.001 compared with the untreated control (0 µM). (Si-TGCT-1: passage 10, Si-TGCT-2: passage 26, Si-TGCT-3: passage 20, and Si-TGCT-4: passage 15)

**Fig. 6:**
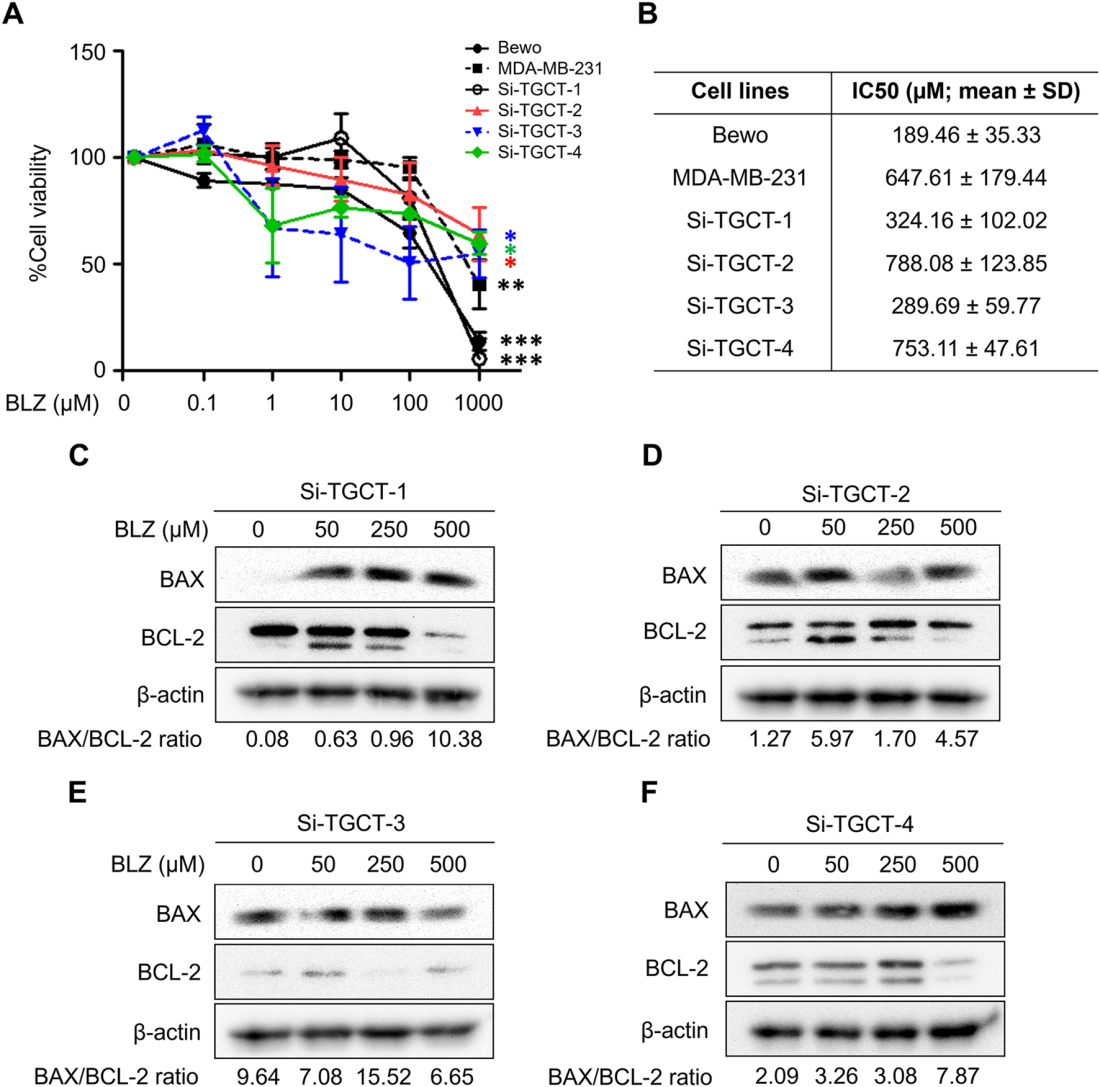
Sotuletinib (BLZ945) induced cell death in TGCT cells. **A** Cytotoxicity analysis of sotuletinib  treatment was performed by MTS assay at 0, 0.1, 1, 10, and 100 µM for 96 h. **B**–**F** TGCT cell pellets treated with sotuletinib at concentrations 0, 50, 250, and 500 µM for 96 h were subjected to check the expression of BAX and BCL‑2 by Western blot analysis. β‑actin was used as the protein loading control. The ratio of BAX/BCL‑2 was analyzed and reported from the relative band intensity of the Western blotting. *P < 0.05; **P < 0.01 and ***P < 0.001 compared with the untreated control (0 µM). Si-TGCT-1: passage 10, Si-TGCT-2: passage 26, Si-TGCT-3: passage 20, and Si-TGCT-4: passage 15)

To determine the relevant toxicities of pexidartinib and sotuletinib, TGCT cells were exposed to increasing concentrations of pexidartinib (Table 3; Fig. 5) and sotuletinib (Table 3; Fig. 6). The concentration of pexidartinib used in the experiment were ranged from 0, 0.2, 2, 20, and 200 µM, and the concentrations of sotuletinib were 0, 0.1, 1, 10, 100, and 1000 µM. Cell viability was assayed at 96 h. Pexidartinib exhibited the lowest IC_50_ values for all cell lines compared with sotuletinib. However, Si-TGCT-2 and Si-TGCT-4, which expressed less CSF1R, demonstrated the highest IC_50_ values. Bewo represented high CSF1R expressing cells, whereas MDA-MB-231 was a low CSF1R cell line.

**Table 3:** Summary of half-maximal growth inhibition concentration (IC50; mean ± SD) for 96 h by MTS assay

TGCT cells	IC50 (µM; mean ± SD) at 96 h	
	Pexidartinib (PLX3397)	Sotuletinib (BLZ945)
Si-TGCT-1	9.61 ± 2.95	324.16 ± 102.02
Si-TGCT-2	152.05 ± 32.74	788.08 ± 123.85
Si-TGCT-3	46.62 ± 8.16	289.69 ± 59.77
Si-TGCT-4	57.60 ± 7.99	753.11 ± 47.61
Bewo	5.64 ± 1.84	189.46 ± 35.33
MDA-MB-231	185.48 ± 48.42	647.61 ± 179.44

### Cell authentication by STR profiling

Fingerprint confirmed novelty of all 4 cell lines compared to STR data from ATCC, DSMZ, JCRB, ECACC, GNE and RIKEN databases (Supplementary data 1). The TGCT fingerprint was identical to that of the white blood cells of the patient whose tissue was used to establish four lines.

## Discussion

While complete synovectomy is a standard treatment for TGCTs, it is often challenging because of the high rate of local recurrence [3, 12, 23]. The United States Food and Drug Administration approved pexidartinib for use with patients with advanced diseases for whom surgical treatment was not feasible [24]. The effectiveness of pexidartinib, a CSF1R inhibitor, has been proven in TGCTs. However, the response rate evaluated by RECIST criteria or tumor volume score has ranged between 39 and 60% [24, 25]. Therefore, a novel treatment is needed. Patient-derived cell lines facilitate discoveries in cancer biology and translational research. Only 2 TGCT cell lines are available from the public cell bank [26, 27]. Considering the genetic diversity of TGCTs, the number of TGCT cell lines remains inadequate. We established four novel TGCT cell lines—Si-TGCT-1, -2, -3, and -4—derived from the primary tumors of patients with TGCTs.

Regarding the backgrounds of the patients from whom the Si-TGCT-1–4 cell lines were sourced, two lines were derived from localized-type TGCTs (Si-TGCT-1 and -2) and two were sourced from diffused TGCTs (Si-TGCT-3, and -4). The original tumors were found in typical locations of TGCT: the knee, ankle, and hip joints. Given the patients’ mean age of 48 (range, 38–70) years and their variety of TGCT types and localizations, the Si-TGCT-1–4 cell lines were derived from patients with clinical features dissimilar to those of patients used to source previously established TGCT cell lines.

The morphology of the Si-TGCT-1–4 cells was mainly spindle- and polygonal-shaped under culture conditions in both the two-dimensional and spheroid forms. The Si-TGCT-1–4 cells showed constant but slow growth. Neoplastic TGCT cells with CSF1 translocation are most likely to recruit CSF1R-expressing macrophages, which may induce the formation of multinucleated giant cells [28]. Regarding giant cell appearance during cultivation, we found that the morphology of Si-TGCT-1–4 cells was mainly spindle-shaped. Osteoclast-like multinucleated giant cells were not found in the plate or spheroid of Si-TGCT-1–4, and the major component of the spheroid was the spindle. Since HE-stained tumor tissues of TGCT contain multinucleated cells, the Si-TGCT-1–4 cell lines are clonal cell lines of the spindle cell population. The Si-TGCT-1–4 cells also formed spheroids on a low attachment substrate. Thus, they can be used to examine the effects of complex architecture on drug sensitivity. Although, one cell (Si-TGCT-1), the localized type, showed slower two-dimensional-growth, the rest of TGCT cells, both localized and diffused type showed no differences in their two-dimensional-growth or spheroid-growth abilities.

Translocations involving chromosome 1p13 are present in a majority of cases of TGCTs. However, only approximately 30% of cases, *CSF1* is fused to *COL6A3* (2q35) [29, 30]. Overexpression of *CSF1* occurs only in a minority of the TGCT cells, whereas most cells express *CSF1R*, not *CSF1*. [5, 6]. Similar results were obtained in the Si-TGCT-1–4 cells in that CSF1R was highly expressed, whereas *CSF1* was rarely found on immunofluorescence staining and Western blotting (Table 2; Fig. 3). All four cell lines did not show *CSF1-COL6A3* fusion on the short tandem assay (STR), which may represent most of the TGCTs.

In our drug testing, pexidartinib reduced the proliferation of Si-TGCT-1–4 markedly better than sotuletinib for all cell lines, with sotuletinib’s IC50 values being 5–33 times higher than those produced with pexidartinib. Notably, Si-TGCT-1, and -3 showed the highest sensitivity to pexidartinib by their CSF1R affinity. The BAX/BCL-2 ratio increased with pexidartinib and sotuletinib treatment. As a tyrosine kinase inhibitor, pexidartinib targets CSF1R, *KIT*, *FLT3*, and platelet-derived growth factor receptor-β. These are receptor tyrosine kinases that are involved in regulating critical processes within cells. Sotuletinib is a highly effective, selective, and brain-penetrating inhibitor of CSF-1R (c-Fms). Clinical trials of two tyrosine kinase inhibitors with activity against CSF1R, imatinib [31] and nilotinib [32], have shown fair response rates, as have the CSF1R monoclonal antibody emactuzumab [14, 33]. Along with clinical studies, there is a need to identify more effective and less toxic drugs or drug combinations added on to pexidartinib to treat TGCTs based on in vitro studies.

In conclusion, we established novel TGCT cell lines, Si-TGCT-1–4, which exhibited continuous proliferation and spheroid formation. We identified CSF1R in each cell line, with different expression levels among cells. We also added the antitumor effects of pexidartinib and sotuletinib on TGCT cell lines, which worked according to how much CSF1R they had. The results show that Si-TGCT-1–4 cells have the potential to facilitate numerous advances in preclinical and basic research on TGCTs.

## Supplementary Information

Below is the link to the electronic supplementary material.

Supplementary file1 (DOCX 5089 KB)

## Data Availability

Cell lines and data are available upon request.

## Abbreviations

α‑SMA: α‑Smooth muscle actin
BLZ945: Sotuletinib
CSF1: Colony-stimulating factor 1
CSF1R: Colony-stimulating factor 1 receptor
CK-19: Cytokeratin-19
FAP: Fibroblast activation protein
panCK: Pan cytokeratin
PLX3397: Pexidartinib
TGCT: Tenosynovial giant cell tumor
