# Supplementary material for: Novel CSF1R-positive tenosynovial giant cell tumor cell lines and their pexidartinib (PLX3397) and sotuletinib (BLZ945)-induced apoptosis
Source: Hum Cell. 2022 Dec 2;36(1):456–67. doi: 10.1007/s13577-022-00823-0 (PMC9813176; doi:10.1007/s13577-022-00823-0)
Supplement: Supplementary file 1 — Supplementary file1 (DOCX 5089 KB) [file 13577_2022_823_MOESM1_ESM.docx]

**Supplementary data 1** Fingerprints of the TGCT-01, -02, 03 and -04 cell lines.

| STR Locus | Allele(s) | | | |
| --- | --- | --- | --- | --- |
|  | TGCT-01 | TGCT-02 | TGCT-03 | TGCT-04 |
| Amelogenin | X,Y | X,Y | X,X | X,Y |
| D3S1358 | 16,18 | 15,15 | 15,16 | 15,16 |
| D1S1656 | 13,15 | 14,18.3 | 15,16 | 15,17 |
| D2S441 | 12,15 | 11,12 | 11,12 | 11.3,15 |
| D10S1248 | 13,16 | 13,16 | 12,14 | 16,17 |
| D13S317 | 8,13 | 9,9 | 8,8 | 11,12 |
| Penta E | 11,18 | 11,11 | 12,14 | 12,14 |
| D16S539 | 9,10 | 11,12 | 13,13 | 11,12 |
| D18S51 | 12,18 | 15,16 | 13,16 | 15,15 |
| D2S1338 | 17,20 | 19,23 | 20,20 | 17,18 |
| CSF1PO | 12,13 | 11,12 | 11,13 | 10,12 |
| Penta D | 9,13 | 8,9 | 9,10 | 9,10 |
| TH01 | 9,9 | 6,9 | 7,10 | 9,9 |
| vWA | 18,19 | 14,19 | 14,18 | 14,18 |
| D21s11 | 30,31.2 | 29,33.2 | 30,30 | 32,33.2 |
| D7S820 | 11,11 | 9,11 | 11,12 | 10,11 |
| D5S818 | 10,10 | 12,13 | 10,13 | 11,13 |
| TPOX | 8,11 | 8,8 | 8,10 | 8,11 |
| D8S1179 | 13,15 | 10,14 | 12,14 | 13,14 |
| D12S391 | 18,20 | 19,19 | 19,21 | 15,18 |
| D19S433 | 12,15.2 | 14,16.2 | 14,14 | 15.2,16.2 |
| SE33 | 28.2,28.2 | 22,31.2 | 19,21 | 21,28.2 |
| D22S1045 | 11,11 | 16,16 | 16,16 | 11,17 |
| DYS391 | 10 | 10 | - | 10 |
| FGA | 21.2,24 | 19,23 | 24,26 | 22,23 |
| DYS576 | 20 | 21 | - | 17 |
| DYS570 | 17 | 17 | - | 20 |

**Cell authentication by STR profiling** Fingerprint results confirmed novelty of these 4 cell lines. The TGCT fingerprint was identical to that of the white blood cells of the patient whose tissue was used to establish four lines (data not shown).
